# High-precision density mapping of marine debris and floating plastics via satellite imagery

**DOI:** 10.1038/s41598-023-33612-2

**Published:** 2023-04-26

**Authors:** Henry Booth, Wanli Ma, Oktay Karakuş

**Affiliations:** 1 School of Computer Science and Informatics, Cardiff University, Abacws, Cardiff CF24 4AG UK; 2 Met Office, FitzRoy Road, Exeter, Devon EX1 3PB UK

**Keywords:** Environmental impact, Environmental sciences

## Abstract

The last couple of years has been ground-breaking for marine pollution monitoring purposes. It has been suggested that combining multi-spectral satellite information and machine learning approaches are effective to monitor plastic pollutants in the ocean environment. Recent research has made theoretical progress in identifying marine debris and suspected plastic (MD&SP) through machine learning whereas no study has fully explored the application of these methods for mapping and monitoring marine debris density. Therefore, this article consists of three main components: (1) the development and validation of a supervised machine learning marine debris detection model, (2) to map the MD&SP density into an automated tool called *MAP-Mapper* and finally (3) evaluation of the entire system for out-of-distribution (OOD) test locations. Developed MAP-Mapper architectures provide users with options to achieve high precision (*abbv.* -HP) or optimum precision-recall (*abbv.* -Opt) values in terms of training/test dataset. Our MAP-Mapper-HP model greatly increases the MD&SP detection precision to 95%, while the MAP-Mapper-Opt achieves 87–88% precision–recall pair. To efficiently measure density mapping findings at OOD test locations, we propose the *Marine Debris Map* (MDM) index, which combines the average probability of a pixel belonging to the MD&SP class and the number of detections in a given time frame. The high MDM findings of the proposed approach are found to be consistent with existing marine litter and plastic pollution areas, and these are presented with available evidence citing literature and field studies.

## Introduction

Current estimates indicate that there are now millions of tons of plastic floating in the world’s oceans, with millions more entering each year^1^. Since plastics have an extremely slow rate of decomposition, marine plastic is rapidly accumulating. Whilst micro-plastics (0.05–0.5 cm) and mesoplastics (0.5–5 cm) are by far the most numerous, macro (5–50 cm) and mega-plastics (>50 cm) are thought to make up the majority of the total weight of ocean plastic^1^. Therefore, effective monitoring and mapping of larger plastic objects are needed to answer key scientific questions regarding the sources, distribution, and transportation of plastic in the ocean environment. These insights could help advise preventative measures, clean-up operations, and improving their efficacy^2^. In recent years, machine learning techniques have been successfully applied for classification in various vision-related areas such as healthcare, text analytics, cybersecurity, and geo-sensing problems such as road extraction and land cover mapping^3–5^. Furthermore, advances in this field have led to models that can outperform human experts in some tasks ^6,7^. Machine learning algorithms can therefore remove the need for manual labelling, whilst maintaining and sometimes improving performance.

The aforementioned technical advance, hence, opens gates for efforts to automatically detect and classify marine plastic via machine learning and computational imaging algorithms with the capability to examine large areas for plastic pollution^8,9^. In an initial study, Aoyama^10^ developed a method for identifying marine plastic by using two-dimensional scatter diagrams of satellite spectral bands. Pixels that had large spectral differences from the surrounding ocean were suspected to be plastic. The method was then validated using a known target of a fixed fishing net with buoys. Furthermore, in the 2019 stage of the Plastic Litter Project (PLP^11^), Topouzelis et. al. ^12^ suggested investigating three artificially created plastic targets in Greece. The objective of this work was to assess the spectral signatures of plastic targets in Sentinel-2 satellite imagery. It was suggested that these findings would help assess which wavelengths offer the best opportunity to differentiate sea foam, white caps, and surface-reflected glint from marine debris.

Thanks to the initiative studies mentioned above^10,12^, the capability of spectral reflectance information to discriminate plastic from other targets has driven further research in this area. Tasseron et. al.^13^ presented a hyper-spectral laboratory setup to collect spectral signatures of 40 virgin macroplastic items and vegetation. Their results returned absorption peaks of plastics (1215 nm, 1410 nm) and vegetation (710 nm, 1450 nm), and provided evidence of the utilised spectral indices such as shortwave infrared (SWIR), and near-infrared (NIR) whilst developing normalised vegetation difference index (NDVI) and floating debris index (FDI) metrics. Knaeps et. al.^14^ proposed literature with a data set of 47 hyperspectral-reflectance measurements of plastic litter samples in dry and wet conditions from the Port of Antwerp. Their results specifically highlighted water absorption and suspended sediments which could allow future research to appropriately select wavelengths. Garaba et. al.^15^ proposed an analysis of the reflectance measurements collected from virgin and ocean-harvested plastics. Their findings showed that ocean-harvested plastics (ropes, foam, etc.) followed identical absorption features and had lower reflectance compared to virgin plastics (low-density polyethylene (HDPE, LDPE), polypropylene (PP)). Moshtaghi et. al.^16^ proposed one of the most important hyperspectral reflectance analyses of plastics in a controlled environment. The authors analysed reflectances of virgin and natural plastics submerged in water with different sediment conditions and depths. Their findings provided evidence to utilise SWIR and visible spectrum for plastic detection.

Parallel with the above-mentioned emerging research on investigating spectral features to discriminate marine debris and floating plastics from others, researchers also investigated the detection potential of aerial and satellite imagery via exploiting spectral analysis. Moy et al.^17^ used aerial surveys and manual inspection of images to map the quantity and location of macro-plastics on the coastline of the eight main Hawaiian islands. They found that windward shorelines had the highest density of plastic and produced a map of the coastline to visualise this. This study demonstrates the ability to map marine plastic on coastlines using aerial imagery. Biermann et al.^18^ demonstrated that it was possible to train a machine learning algorithm to differentiate between plastic and other types of marine debris. This seminal work proposed the FDI—a novel parameter increasing the detectability of suspected plastics via promoting sub-pixel interactions of plastics with the sea surface. Bierman et. al.^18^ used Sentinel-2 multispectral data in their developed models and trained them with several marine debris targets extracted via FDI and NDVI from Scotland, British Columbia, Barbados and Durban. In terms of the validation stages of the proposed approach, this work utilised the plastic targets developed by Topouzelis et al.^12^. They reported a maximum accuracy of 86% for the classification of suspected plastics among other debris such as plumes, timber, and seaweed.

Furthermore, Kikaki et al.^19^ investigated the plastic pollution problem in the Bay Islands of Honduras by using remote-sensing observations from 2014 to 2019. It was noted that detectable plastics generally follow linear patterns. An automated plastic pollution monitoring approach for the river surfaces using bridge-mounted camera imagery was presented by van Lieshout et. al.^20^. The authors performed an experimental analysis of five different rivers in Jakarta, Indonesia with the highest 69% precision of plastic detection . Park et. al.^21^, instead of Sentinel-2, utilised very high geospatial resolution 8-waveband WorldView-3 imagery in order to observe floating plastic litter in the Great Pacific Garbage Patch (GPGP). They applied various spectral analysis approaches and investigated anomalies to infer the presence of suspected plastic litter. Ciappa^22^ proposed a detection approach for marine litter patches from Sentinel-2 offshore Hawaii’s Big Island. The author focused on the discrimination between the sargassum and plastics where the findings suggested that the red-edge spectra were more likely to provide differences between the targets. Kremezi et al.^23^ leveraged the potential of satellite hyper-spectra remote sensing imagery in marine plastic litter detection purposes for the first time in the literature. The authors used PRISMA satellite data with fine spectra but low spatial resolutions. In order to increase the spatial resolution, Kremezi et al.^23^ proposed exploiting pansharpening with the panchromatic data that enhance spatial resolution.

Up to this point, all the literature pieces have been using their own data sets and/or some non-open-access field studies except for a couple of initiatives (PLP^11^) that share their data for further research. However, the available amount of data was not enough to drive marine debris monitoring research to further advanced machine learning stages similar to the one e.g. in computer vision research. Thanks to a recent work published in 2022, marine debris monitoring research obtained its first detailed open-access data set to further develop machine learning approaches. In this milestone study, Kikaki et al.^24^ produced a Marine Debris Archive (MARIDA) data set which contains 1381 patches with 837,357 annotated pixels from 63 Sentinel-2 scenes acquired between 2015 and 2021. The patches are distributed over eleven countries. MARIDA dataset is based on Sentinel-2 multi-spectral satellite data providing 15 thematic classes including (marine debris, dense sargassum, natural organic material, clouds, foam, etc.). MARIDA contains 3339 ( 0.4%) Marine Debris pixels in total which were defined as “floating plastic and polymers, mixed anthropogenic debris”. Of these plastic pixels, 1625 pixels were digitised and annotated with high confidence. This study also investigated the effectiveness of different machine-learning algorithms in detecting marine debris. Three variations of the random forest model were examined, as well as a U-net model where Random Forest models outperformed the U-net model.

When discussing the MARIDA^24^ dataset and the models developed using it, “marine debris” will be used along with the term “suspected plastic” (MD&SP shortly) for the rest of this work. This increases clarity since plastic is thought to make up the vast majority of floating anthropogenic debris^25^. We also believe that this prudent approach in defining the detected pollutants is also aligned with the cautious note on spectral interpretations published by Hu recently^26^. Lastly, the literature covers various other important works which can be seen as less relevant to the approaches reported in this paper. Thus, interested readers are recommended to refer to two newly published review papers^27,28^ for more details on artificial intelligence utilisation in marine litter/plastic detection approaches to further expand the research area.

Consequently, this paper explores an optimised machine-learning method with the primary aim of assessing the feasibility of mapping MD&SP density on multi-spectral satellite imagery. Whilst reaching the aforementioned aim, we (1) develop a machine learning algorithm to enable precise detection of pollutants on the ocean surface, (2) develop an automated data pipeline that is capable of gathering, pre-processing and making predictions on satellite data, (3) use the data-pipeline to generate marine debris density maps for the test locations. To address the aforementioned aims, this work proposes a novel end-to-end automated system, named as MAP-Mapper, consisting of two main components: (i) a high-precision MD&SP detection machine learning algorithm, and (ii) a scientific tool/pipeline to facilitate MD&SP density mapping for any region of interest (ROI) in any given time frame. We evaluate the proposed detection algorithm with the MARIDA data set whilst Topouzelis et. al.^11^’s PLP 2021 data set is used to validate the MAP-Mapper architectures. Six test locations including polluted areas such as Manila - Philippines, and Mumbai - India are used to test the proposed MAP-Mapper system. In order to efficiently quantify the density mapping findings, we propose a pixel-level parameter - the Marine Debris Map (MDM) index, that combines the average probability of belonging to the MD&SP class and the number of detections within the given time interval. High MDM findings of the MAP-Mapper algorithms have been found to be aligned with the existing marine debris and plastics pollution areas, and these are presented with existing evidence referring to the literature and field studies.

## Results

### Model evaluation with MARIDA data set

Baseline models provided by Kikaki et al.^24^ used to test the MARIDA data set had significant problems in terms of run-time and/or precision. Particularly, Kikaki et al.^24^ models had a tendency to misclassify targets such as marine water, sea foam, ships, and clouds as suspected plastics. This raises a problem of low precision in terms of performance for future applications since the miss-classified targets generally accommodate together in a natural sea environment. The aforementioned fact can be seen as evidence that shows the importance of the high-precision models^29^. Low precision is likely to result in significant numbers of false positives. Consequently, current models lack the precision required and are likely to produce inaccurate MD&SP density maps.

To provide a solution to the aforementioned problem, we conducted a development procedure of multiple U-net-based machine learning models to produce an optimised model and enable more accurate and high-precision density maps. Kikaki et. al. ^24^ MARIDA dataset was used to train and evaluate the proposed models. Particularly, we developed two different MAP-Mapper models to provide (1) high precision (*abbv.* -HP) and (2) theoretically optimum - in terms of precision-recall values—(*abbv.* -Opt). The comparison results and performance metrics for MARIDA data tests are given in Table 1.

**Table 1:** Metrics for plastic detection and model comparison. $^+$ MD&SP refers to Marine Debris and Suspected Plastics.

Model	MD&SP$^+$			Overall		
	mIoU	Precision	$F_1$ -score	mIoU	Precision	$F_1$-Score
Kikaki et al.^24^ U-net	0.33	0.35	0.49	0.66	1.00$^*$	0.74
Kikaki et al.^24^ RF	0.67	0.79	0.83	0.70	N/A	0.81
MAP-Mapper-Opt	**0.78**	0.87	**0.88**	**0.89**	1.00$^*$	**0.94**
MAP-Mapper-HP	0.60	**0.95**	0.75	0.80	1.00$^*$	0.88

$^*$ Due to the large class imbalance, the overall precision of all classification models were close to, and thus rounded to, 1.00, regardless of other metrics. Best performing model values are shown in bold.

**Figure 1:**
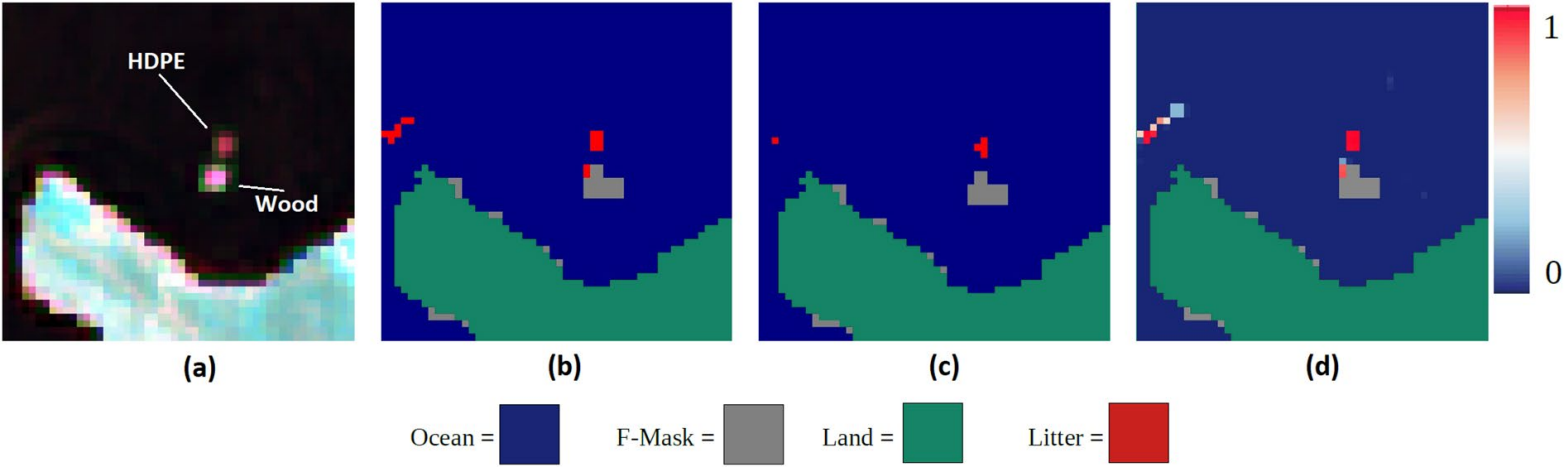
Gulf of Lesvos suspected plastic detection. (**a**) Sentinel-2 false colour image, (**b**) MAP-Mapper-Opt, (**c**) MAP-Mapper-HP, (**d**) Probability map. The scene includes two targets covering an area of more than 600 m2. The original work did not state the exact number of target pixels since the aim was to guarantee at least one %100 plastic pixel. From the visual analysis, both targets cover around 9 pixels (6 of which with a high reflectance return) shown in (**a**). The colour bar on the right corresponds only to subfigure (**d**) and presents probability values of pixels where green is the land and grey ones are F-masked pixels. For the model in (**b**), 10 pixels of the HDPE and 6 of the wooden were classified correctly. 4 out of 6 of these pixels were then masked by F-mask. A number of false positives can be seen on the left of the figure, which appears to be the result of a ship or wake. For the model in (**c**), 4 HPDE pixels were classified as plastic, whilst no pixels from the wooden target were classified. 1 false positive was seen at a threshold of 0.99.

### Model validation with PLP 2021 data set

The Island of Lesvos, Greece has been the site of the Plastic Litter Project since 2018. The experiment conducted by the University of Aegean’s Marine Remote Sensing Group in 2021 was selected for validation of the MAP-Mapper tools based on the certainty of the target position, material, and size. This is giving us the chance to explore the strengths and weaknesses of the MAP-Mapper algorithms^11^. For the PLP 2021, two large targets were deployed, both approximately 28 meters in diameter (Fig. 1a). One comprised high-density polyethylene (HDPE) plastic mesh and the other was made of wood. At a threshold of 0.5 in Fig. 1b, 10 pixels of the HDPE target and 6 of the wooden target were classified correctly. 4 out of 6 of these wooden pixels were then masked by F-mask^30^. A number of false positives were observed which appear to be the result of a ship or wake. At a threshold of 0.99 in Fig. 1c, 4 HPDE pixels were classified as suspected plastic, whilst no pixels from the wooden target were classified. 1 false positive was seen at a threshold of 0.99. F-mask appears to mask part of the natural wooden target but did not mask the plastic target. Figure 1d depicts the probability map for this scene. It can be seen that high-probability pixels mostly overlap with the correct plastic pixels with a few low-probability detections on the left side of Fig. 1d.

### MD&SP density mapping

This experiment case enables the assessment of MAP-Mapper model performances in different test regions and their global applicability. Marine debris density maps were produced from data spanning around a year for each test location. By following news about mass pollution events and also data from OpenOceans Global ^31^ and the OceanCleanUp river monitoring software ^32^, we decided on 6 test locations which are (1) the Gulf of Honduras, (2) Manila - Philippines, (3) Mumbai - India, (4) Hong Kong, (5) Cornish coastline, UK and (6) Chubut, Patagonia, Argentina. The first three test locations accommodate potentially high MD&SP coverage and are selected to test proposed approaches in high-density scenarios whilst the remaining three are chosen to test the proposed approaches’ capabilities in locations with no/low MD&SP. The details and data information of each test location is presented in Table 2.

**Figure 2:**
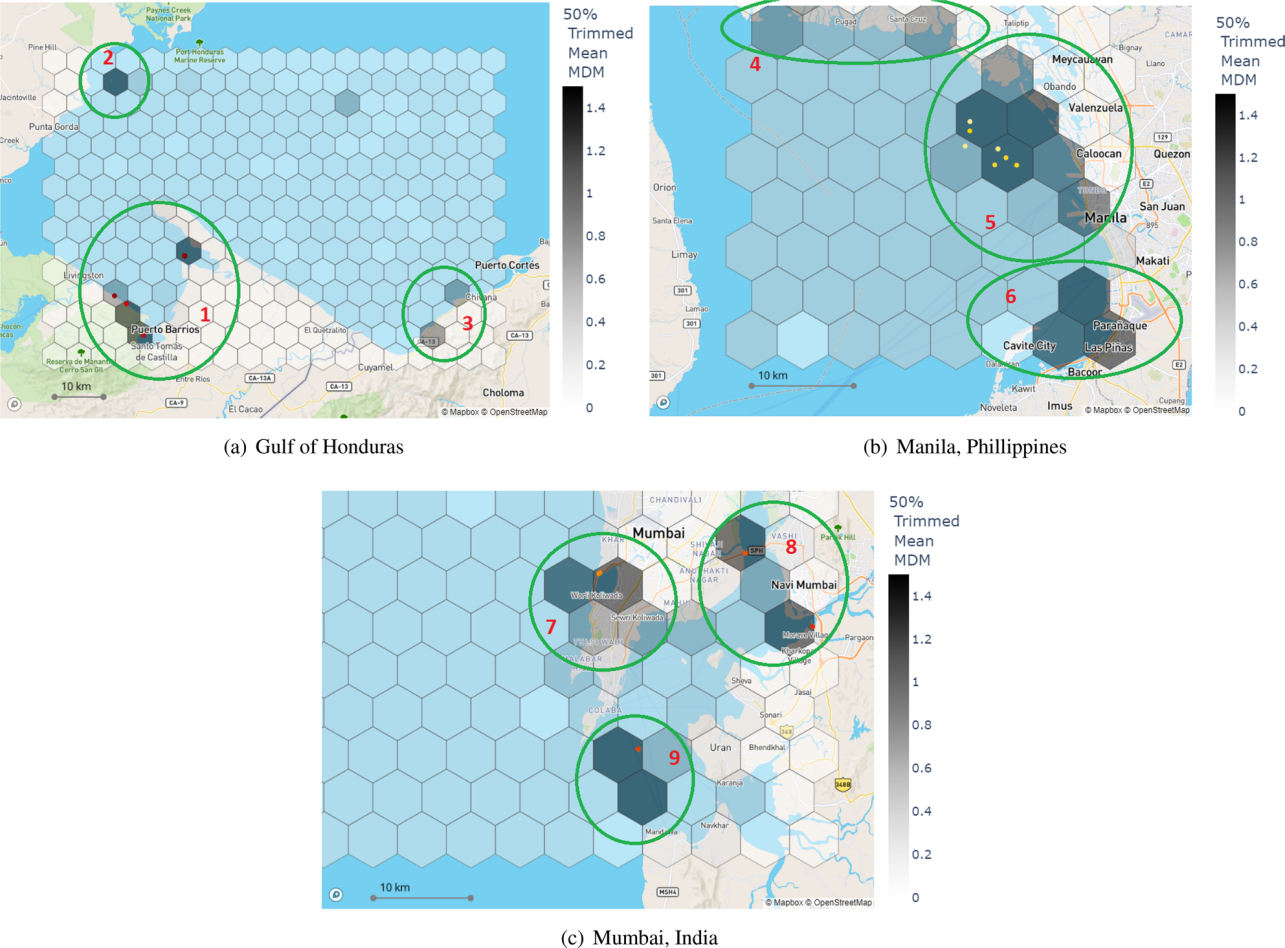
Potentially polluted test location MD&SP density maps. The colour bar corresponds to 50% left-trimmed average MDM values of each hexagon. Each figure also includes the top 10 highest pixel-level MDM values as scatter points where brighter markers correspond to higher MDM values. Each hexagon has a width of 5 km and an area of $\approx$22km$^2$. Each subplot shows south-to-north directions from bottom to top, respectively.

**Figure 3:**
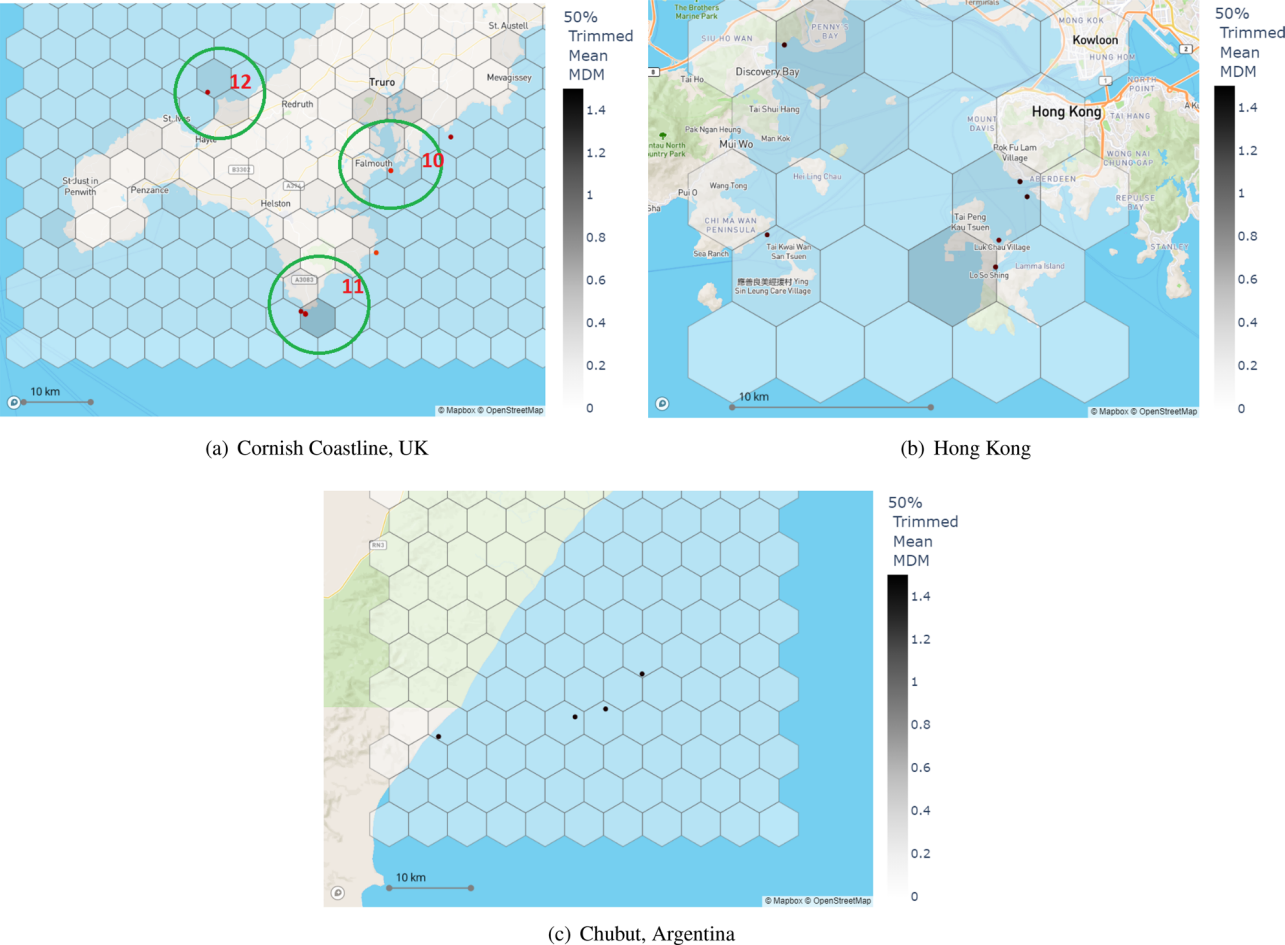
Potentially no- or less-polluted test location MD&SP density maps. The colour bar corresponds to 50% left-trimmed average MDM values of each hexagon. Each figure also includes the top 10 highest pixel-level MDM values as scatter points where brighter markers correspond to higher MDM values. Each hexagon has a width of 5 km and an area of $\approx$22 km$^2$. Each subplot shows south-to-north directions from bottom to top, respectively.

MD&SP density maps for each test location are shown in Figs. 2 and 3. In each figure, the hexagonal width was set to 5km and for all plotted images each hexagonal area was fixed at approximately 22 km$^2$. Colour coding in each hexagon shows 50% left trimmed average (values lower than the median of each hexagon removed and not included for average calculations) MDM values considering each hexagon includes a multitude of pixels having 0 or small (up to 0.2) MDM values. This enables us to remove pixels that do not contribute to the density analysis in each hexagon and to better quantify the novel MDM metric for the purposes of MD&SP density mapping. For each test location, we also displayed the top 10 highest MDM values as scatter points.

**Table 2:** Details and data information of each test location evaluated for MD&SP density mapping.

Test location	Date interval	# of dates	Location coordinates	Area (km$^2$)
Bay of Honduras	1 Jan 2022	25	(16$^\circ$11$'$56$''$N , 88$^\circ$48$'$29$''$W)	5221.87
	13 Sep 2022		(15$^\circ$40$'$38$''$N , 87$^\circ$58$'$03$''$W)	
Cornish coastline, UK	12 Jan 2022	30	(50$^\circ$31$'$13$''$N , 5$^\circ$48$'$03$''$W)	6955.98
	26 Oct 2022		(49$^\circ$56$'$39$''$N , 4$^\circ$17$'$05$''$W)	
Hong Kong	4 Jan 2022	37	(22$^\circ$21$'$11$''$N , 113$^\circ$59$'$48$''$E)	320.96
	26 Oct 2022		(22$^\circ 11^{\prime }42^{\prime \prime }N$, 114$^\circ$10$'$26$''$E)	
Manila, Philippines	7 Jan 2022	15	(14$^\circ$51$'$25$''$N , 120$^\circ$39$'$13$''$E)	1605.43
	3 Nov 2022		(14$^\circ$30$'$03$''$N, 121$^\circ$01$'$51$''$E)	
Chubut, Patagonia, Argentina	9 Jan 2022	44	(45$^\circ$13$'$07$''$S, 67$^\circ$37$'$08$''$W)	5846.64
	28 Oct 2022		(45$^\circ$58$'$30$''$S, 66$^\circ$43$'$13$''$W)	
Mumbai, India	2 Jan 2022	21	(19$^\circ$33$'$43$''$N, 72$^\circ$30$'$08$''$E)	5574.59
	3 Nov 2022		(18$^\circ$39$'$31$''$N, 73$^\circ$01$'$54$''$E)	

#### Gulf of Honduras

The Gulf of Honduras is an important inlet in the Caribbean Sea including coastal areas of Honduras, Guatemala and Belize. Even though it is seen as one of the important spots of a diverse and unique ecosystem due to the effects of strong ocean currents, the Gulf of Honduras is also one of the well-known plastic hotspots that have been the research site of numerous studies investigating marine-plastic pollution.

The MAP-Mapper density mapping results highlight three important regions in this test location. The first area is in the Bay of Amatique. Most of the detections appear to be contained within a relatively small area (4 hexagons in total). This location was identified as the coasts of Macho Creek, Puerto Barios, and Bahia La Graciosa. This area contains all the highest MDM-valued locations and has an average MDM of around 2.45 with maximum MDM values of around 7.00 (1 in Fig. 2a). The second region (2 in Fig. 2a) is off the coast of Punta Gorda. There is one hexagon with an average MDM value of 1.52. The third area of high MD&SP density is noted off the coasts of Omoa, near the Motagua river mouth, which is located southwest of Omoa. This area has two dense hexagons with average MDM values of around 1.00 (3 in Fig. 2a).

#### Manila—Phillipines

Manila is the capital city of the Philippines sitting in the metropolitan area of Metro Manila which is the 5$^{th}$ populous metropolitan area in the world. Manila has also a negative reputation for having a pollution problem and is another important globally acknowledged MD&SP hotspot via accommodating three out of five most pollutant rivers in the world^31^.

MAP-Mapper density mapping results are highlighted under three regions as given below. The first region is Manila centre and its north coastlines. In this area, 6 hexagons ($\approx$130km$^2$) are having average MDM values higher than 1.00 (with the highest 2.87). All 10 highest pixel-level MDM values recorded within this area values of which are changing between 9.00 and 17.00 (5 in Fig. 2b). Cavite City having the highest average MDM value of 3.55 includes 3 hexagons higher than 1.00 MDM (6 in Fig. 2b). Compared to the other two regions highlighted above, the third region has less MD&SP coverage however worth highlighting here. There are 2 densely polluted hexagons off the coasts of San Pascual, and 1 off the coast of Santa Cruz with average MDM values around 0.90 (4 in Fig. 2b).

#### Mumbai—India

Mumbai is the capital city of Maharashtra state of India and named the 8th highest-populous city in the world. Mumbai is known as polluted in terms of its seashores and inland waters especially due to having the world’s 3rd most polluted river of Ulhas.

The MAP-Mapper density mapping findings can be summarised under three regions: The most MD&SP problematic area of Mumbai is the south coasts of the main Mumbai Island coasts of Arabian Sea near Mahim Bay, Mumbai Harbour of Thane Creek, and Back Bay. This area has 4 densely polluted hexagons valued over 1.00 with the highest of 2.40 (7 in Fig. 2c). The second area is inside the city consisting of Navi Mumbai coasts, Panvel creek, and Thane Creek with three high average MDM hexagons with a maximum of 2.05 (8 in Fig. 2c). The mouth of Thane creek opening to the Arabian Sea off the coasts of Uran at the south of Mumbai includes two high MDM hexagons both of which are higher than 2.00 MDM with a highest of 2.77 (9 in Fig. 2c).

#### Cornish coastline, UK & Hong Kong & Chubut, Argentina

The Cornish coastline is located in the Southwest of England, also known as the Cornwall coastline. This area is one of the most tourist-visiting regions in the UK and is known for its outstanding natural beauty. A recent study has indicated that the Cornish coastline is the most plastic-polluted coastline in the UK^33^. However, the severity of the problem is far less than in the aforementioned test locations. Hong Kong is a special administrative region in South China, that can be listed as one of the most densely populated regions in the world. Despite its relatively smaller area of land, Hong Kong is one of the most important regions in the world in terms of import/export traffic. Chubut province in South Argentina, located in the Andes mountains to the west and the Atlantic Ocean to the east, is one of the important wildlife tourist places in the world especially via accommodating one of the globally largest Magellanic Penguin breeding areas.

All these three aforementioned test locations are sharing the same basis in terms of marine pollution that historically either no or low mass marine pollution problems reported in these areas, and all three test locations are going to be used to test the MAP-Mapper in no/low-density MD&SP coverage. We summarise the MAP-Mapper findings for these test locations as:

- Density maps of all three locations presented in Fig. 3 show similar characteristics as expected. Nearly all the hexagonal regions have brighter colour coding that corresponds to no/low MD&SP density with MDM values less than 0.5.
- Chubut’s highest pixel MDM values are also low and close to 0.00 whilst Hong Kong has some values around 1.50 MDM.
- Even though it, in general, shows low MDM and MD&SP density mapping findings, the Cornish coastline data has some high pixel-level MDM detections ($\approx$7.00), especially near Falmouth estuary (10 in Fig. 3a), that appears to coincide with large quantities of boats at their moorings.

## Discussion

Previous work in the marine debris detection research area has demonstrated that detecting MD&SP using satellite data and machine learning is possible. However, the capabilities of these approaches have not yet been explored on how they can be applied to real-world data for the purpose of automated mapping of marine debris density. With the development of the MARIDA data set^24^, it has now been possible to train new machine learning models and evaluate their effectiveness in density mapping. Consequently, we developed an automated tool—*MAP-Mapper*—to assess suspected plastic concentration and to highlight patterns of debris distribution, and identify key areas of aggregation.

Both *-Opt* and *-HP* versions of the MAP-Mapper obtained considerable results and performance improvements compared to the MARIDA baseline models as seen in Table 1. In particular, MAP-Mapper models improved the MD&SP detection performance up to 16% and 5% in terms of the precision and $F_1$-Score metrics compared to the Kikaki et. al. baseline models. On the other hand, the validation of the Plastic Litter Project targets has been promising. These results show that the model can detect plastic of different types. Furthermore, at lower thresholds, the model is able to detect plastic on the sub-pixel scale. It is unsurprising that a few plastic pixels were detected at a threshold of 0.99. Man-made targets often only covered a small percentage of a pixel. For the larger target, much higher probabilities were assigned to pixels containing 100% plastic. Additionally, the targets were made of a single type of plastic and contained no other types of debris. In the marine environment, plastic patches are extremely unlikely to be this uniform. They usually consist of many different types of plastic and/ or a mix of natural organic material^18,34^. For this reason, the training data is likely to follow this pattern and thus, spectral characteristics of the training data and plastic targets differ to some degree. Finally, the shape and size of the targets are significantly different from the plastic patches in the MARIDA dataset. These contextual factors may help explain why many of the plastic pixels are not predicted as plastic when using high thresholds.

Furthermore, our analysis demonstrates that F-mask has a tendency to mask sargassum patches. Presumably, this is because its spectral signature is similar to terrestrial vegetation, and this is masked as land. Considering plastic is commonly found embedded within sargassum patches ^34^, this was thought to be a potential problem that can cause mask MD&SP pixels. For this reason, we further analysed the prediction files before and after masking and concluded that F-mask did not mask any pixel that was identified as MD&SP. This is a promising finding which suggests that the F-mask is surely a suitable algorithm for plastic detection by not masking MD&SP pixels.

Despite this promising performance of the proposed approaches, manual inspection is needed to be conducted to investigate areas of high plastic density and verify the results of the out-of-distribution test locations where possible. This would help ascertain the strengths and weaknesses of the proposed models. The Bay of Amatique (1 in Fig. 2a) in the Gulf of Honduras was found to have a high density of MD&SP in two different areas as shown in Fig. 2a. The coast off Macho Creek between Livingston and Puerto Barrios is one of the potential MD&SP gathering points for the litter coming out of Rio Dulce that is the source of 16 million kg of mismanaged plastic per year ^32^. The Bay of Amatique has an offshore gyre that rotates counterclockwise and potentially carries the Rio Dulce and other sources of debris to the bays of Puerto Barrios and Bahia La Graciosa. Apart from the Bay of Amatique, Punta Gorda (2 in Fig. 2a) near the Maya Mountains Marine Corridor and the coasts of Omoa (3 in Fig. 2a) appeared as the densely polluted areas which follow academic research for these areas ^35^. With being home to 4 of the world’s 6 most polluted rivers, Manila of the Philippines has become the highest densely MD&SP polluted place in MAP-Mapper analysis. North shores of Manila (4 in Fig. 2b) show some pollution problems with average MDM values of approximately 0.9. MAP-Mapper highlighted the mouths of the world’s first two highest-polluted rivers of Pasig and Tullahan (5 in Fig. 2b). These two rivers are responsible for 550 and 96 million kilograms of mismanaged marine plastic pollutants, respectively ^32^. Moreover, Cavite City near the Freedom Islands (6 in Fig. 2b) has the highest average MDM in the analysis one of the reasons for which is the Paranaque River. This river and its branches are responsible for 10 million of kg mismanaged marine plastic pollutants ^32^. Similarly to the Gulf of Honduras and Manila results, the proposed MAP-Mapper density mapping tools achieved realistic detection results in Mumbai, India test location. Mahim Bay (7 in Fig. 2c) with one of the highest average MDM-valued places in the analysis, also known to be one of the most polluted bays in the world. The world’s third plastic-polluted river Ulhas is also located in Mumbai, and the mouth of this river which is opened to Thane and Panvel Creek (8 in Fig. 2c) is also mapped as a polluted area by the MAP-Mapper tools with MDM values higher than 2.00. South coasts of Mumbai opening to the Arabian Sea are also detected by the MAP-Mapper (9 in Fig. 2c) with two hexagons with MDM approximately of 3.00. The high MDM prediction for this area is expected since all the polluted rivers mentioned above for Mumbai reach the Arabian sea at this mouth.

All the aforementioned explanation shows us that the developed MAP-Mapper tools are realistic and generalisable to out-of-distribution data. The results also suggest that MAP-Mapper Tool is clearly useful for mapping MD&SP density in areas of high pollution. In these regions, the presence of false positives is far less detrimental to the overall density map. True positives appear to outnumber false positives in each of the three polluted test locations above, thus suggesting that MAP-Mapper is a useful tool for mapping the MD&SP density regions like this. In order to show MAP-Mapper’s applicability in regions with known lower plastic pollution, we investigated Cornish Coastline, UK, Hong Kong, and Chubut, Argentina. The results presented in the previous section for these regions show that MAP-Mapper maps these regions with relatively low mean MDM values. Considering the regions of interest for each test location historically do not have important mass MD&SP pollution events, this is parallel with our results and provides shreds of evidence for the global applicability of the MAP-Mapper tools. Cornish Coastline somewhat diverges from Hong Kong and Chubut in terms of pixel-level MDM detections. Both of these regions have the highest pixel level MDM values of around 1.00 whilst Cornish Coastline has values higher than 5.00. The detailed analysis in this region suggests that these pixels are most likely waves and/or sea foam. These features follow the linear trajectory that is typical of the marine debris pixels in the MARIDA dataset but the prevalence leads to the conclusion that some of these detections are likely to be false positives. Although it is not possible to verify every pixel, it is likely that the MARIDA dataset does not contain enough examples of sea foam (0.15% of the whole data set) for the model to differentiate effectively between foam/waves and plastic in this region. Another clear example of miss-classification is found in the Falmouth estuaries (10 in Fig. 3a). Some of the small boats are potentially misclassified as MD&SP. Interestingly, larger boats with wakes were not misclassified. This suggests that more training data is needed to improve model performance when differentiating MD&SP from some types of static watercraft. The results for Cornish Coastlines, especially for pixel level detections around the Lizard (11 in Fig. 3a), St. Ives & St. Agnes (12 in Fig. 3a), can also be read as evidence to the work by Nelms et. al. ^33^ stating that the beaches of these regions are potentially the highest litter accommodating beaches in England.

Table 3 show eight example regions that MAP-Mapper models recognised as high MD&SP density problems with their corresponding average and highest MDM values. The ”Evidence” column in Table 3 refers to references of news/blog pages including real photographs of MD&SP problems in each of these ROIs, and provides evidence regarding the generalisation capabilities of the proposed MAP-Mapper models. The importance of these representations is that, even though the test data is coming from out-of-distribution locations, high-valued MDM density map locations are aligned with the real field studies.

**Table 3:** Some regions recognised as high MD&SP density by the MAP-Mapper models.

Test Location	Location details	Avg MDM	Highest MDM	Position in Figs. 2 and 3	Evidence
Gulf of Honduras	A beach near Livingston, Guatemala	1.87	3.83	Area 1	Ref^36^
	Punta Gora - Maya Mountain Marine Corridor	1.51	3.18	Area 2	Ref^35^
	Off the coasts of Omoa	0.96	3.21	Area 3	Ref^37^
Manila, Philippines	Coasts of Freedom Island	2.90	9.15	Area 6	Ref^38^
	Pasig River	1.90	16.29	Area 5	Ref^39^
Mumbai, India	Mahim Bay	2.40	9.43	Area 7	Ref^40^
	Panvel Creek	2.07	8.89	Area 8	Ref^41^
Cornish Coastline, UK	Polurrian beach on the Lizard Peninsula	0.43	6.99	Area 11	Ref^42^

MAP-Mapper is an initial product from a detailed remote sensing computational imaging project for the purpose of detecting and tracking MD&SP. Our next steps to improve on MAP-Mapper Tool(s) would include (non-exhaustive):

(1) In order to improve the global applicability of MAP-Mapper and to address issues with potential miss-classifications, we plan an expansion of the MARIDA dataset particularly from under-represented regions and predominantly focus on the inclusion of more plastic, foam, ships/boats, and cloud pixels, as well as pixels affected by sun glint. The current version of MAP-Mapper algorithms’ accuracy seriously depends on training data. Due to the limited and unbalanced training data mentioned above, the network is prone to showing less performance for some parts of the world such as the arctics and icy locations. In addition, compared to the open sea locations, the proposed version of MAP-Mapper algorithms (due to MDM usage instead of detections) is more suitable for detecting MD&SP gathering points such as inner waters, and coastal and shoreline places, rather than their trajectories.

MDM currently depends on the percentage of detections in the selected time interval. For scenes that have a high number of images as in the examples in this paper, MDM provided to-the-point evaluation values. However, for scenes having a low number of images (e.g. 4-5) in the given time interval, this might make MDM hard to compare with other scenes with a high number of images. This case is currently under investigation and planned to be improved in future releases.

(2) MAP-Mapper was designed to provide insights into the concentration, distribution, and pathways of marine plastic. Therefore, to help enhance scientific analysis, historical weather queries will also be integrated into the analysis stage. This would make it possible to investigate how recent rainfall correlates with riverine plastic output, especially in locations with poor waste management, e.g. around the mouths of River Motagua in Guatemala and River Pasig in Manila.

(3) MAP-Mapper would benefit from a better assessment of its validity for MD&SP density mapping. To achieve this, suspected plastic detections should be explored with very high-resolution satellite data, ground truth reports, or aerial imagery. This would help certify that pixels are not being miss-classified and help to determine their validity.

(4) The model trained with only 4 bands (the ones contributing to calculating FDI and NDVI) demonstrated good performance than using all 13 Sentinel-2 bands. However, it is possible that other band combinations could improve model performance. Removing some of the lower resolution bands, as well as bands where wavelengths do not correlate with plastic materials, may reduce noise in the data set. Development of future models should trial different band combinations to assess which bands provide the best performance, alongside run-time reduction.

(5) As suggested by Chuanmin Hu^26^ in their cautious note on spectral interpretations, spectrally distorted shapes have an important impact on the visibility of suspected plastics from multi-spectral satellite imagery. For this, we plan to conduct an investigation on the validity of satellite imagery spectral returns with in-situ real marine debris and floating plastics targets.

(6) From a technical point of view, similar to MARIDA baseline models, the MAP-Mapper architectures utilise supervised learning approaches. These are dependent on the labelled training data, and any problem in the training data set, such as low confidence labels or greatly unbalanced class distribution might cause wrong classifications. In the following versions of MAP-Mapper tools, we promote using minimal-supervision (semi-, self- and/or un-supervised) approaches that exploit the usage of unlabelled data to extract further useful information.

## Methods

### Measuring density with a marine debris metric

Developing a density map only taking into consideration the number of detected MD&SP pixels is not suitable considering pixel probabilities in single-date imagery might be high due to several reasons: e.g. misclassifications, dynamic nature of MD&SP, etc. A combination of this with the long time interval and the high number of images for each ROI will strongly affect the accuracy of the MD&SP density maps and create wrong meanings. In order to solve this problem—in the context of density mapping in a time interval—we develop a metric that promotes average pixel probabilities and detection threshold values:

1 $$\begin{aligned} MDM_{ij}&= \left[ \dfrac{100}{N}\sum _{k=1}^N \mathbbm {1}_{p_{ijk}\ge T}\right] \times \left[ \dfrac{1}{N}\sum _{k=1}^N p_{ijk}\right] \end{aligned}$$

2 $$\begin{aligned}&= \mathscr {D}_{ij}\times \bar{\mathscr {P}_{ij}} \end{aligned}$$

where $\mathbbm {1}_{p_{ijk}\ge T}$ is the indicator function that returns 1 when the condition $p_{ijk}\ge T$ is satisfied, and 0 otherwise. The pixel-level probability of being MD&SP is denoted as $p_{ijk}$ on pixel (*i*, *j*) and date *k* whilst *T* refers to the threshold value to determine whether a pixel is MD&SP or not. Thus, $\mathscr {D}_{ij}$ becomes the percentage of the number of detections within the selected time interval whilst similarly $\bar{\mathscr {P}_{ij}}$ refers to the average probability of being MD&SP on pixel (*i*, *j*) for the given time frame.

From the above description, we can see that $MDM_{ij}$ is a positively valued metric where 0 means no MD&SP problem, and a higher *MDM* value corresponds to a polluted location on the maps. On the other hand, *MDM* can be seen as a metric in which the average probability value of a pixel is positively weighted if the total number of detections is high in that corresponding pixel (e.g. 10 dates out of 20 dates in the time interval means 50%). This is giving us a better picture to measure the MD&SP density maps in a global time scale with the MAP-Mapper approach. *MDM* can also be seen to be a universal metric to compare different locations on the earth considering the utilisation of the fixed area of hexagons and the averaging over the given time interval.

### MAP-Mapper pipeline

**Figure 4:**
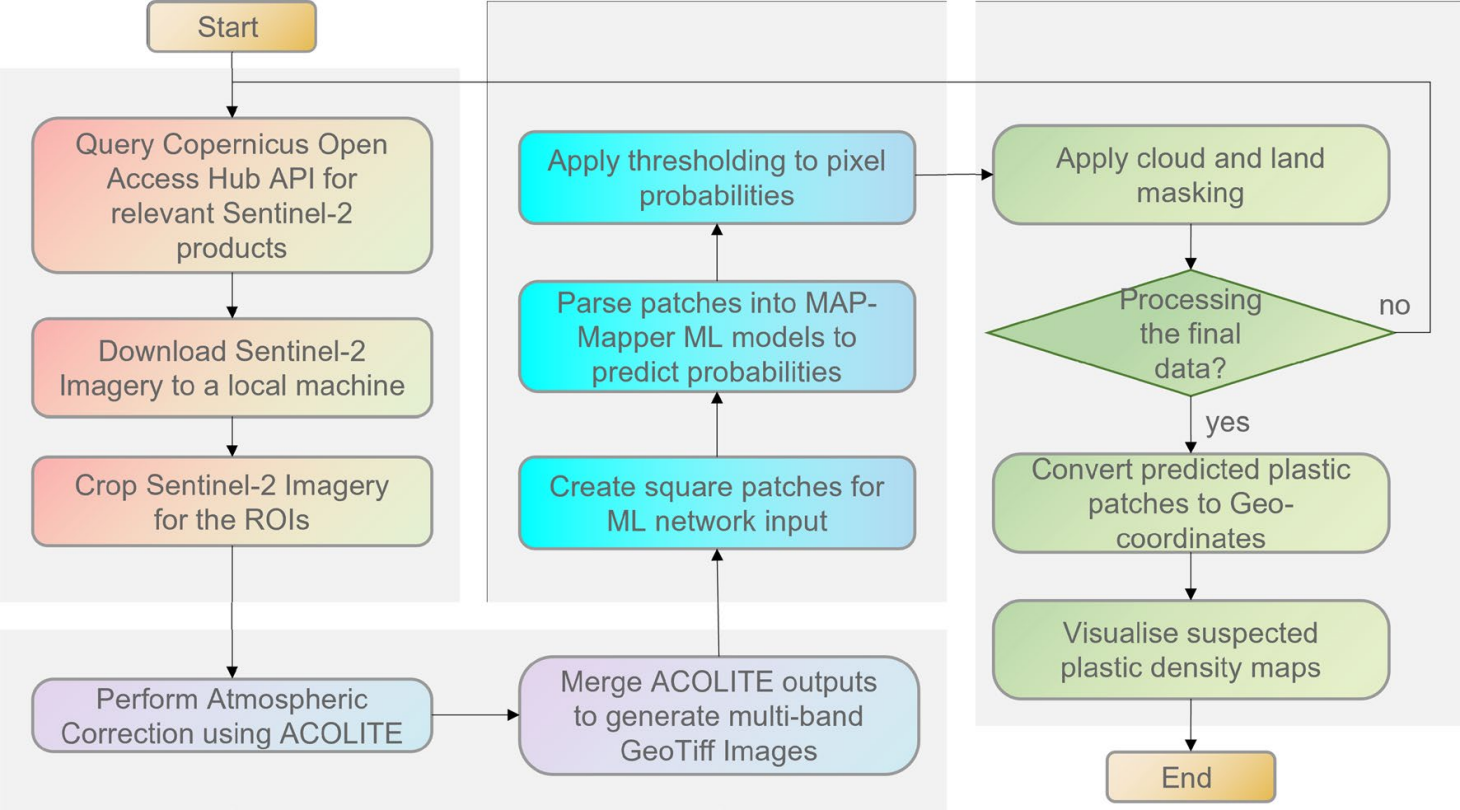
Flow diagram showing the MAP-Mapper pipeline. The above system is fully automated via the Python terminal entering a single command line including the ROI coordinates and several parameters of the network.

The development of the tool was divided into separate components. These components were then integrated and conducted in sequence to produce outputs for the ROI. A flow diagram of this process can be seen in Fig. 4.

*Data gathering* Since the MARIDA dataset consists of Sentinel-2 data, MAP-Mapper requires first to collect Sentinel-2 data for an ROI and date range. This is achieved by querying the Copernicus Open Access Hub API for the required Sentinel-2 data. Querying requires entering corner coordinates of the selected rectangle ROI and desired time interval. MAP-Mapper then downloads Sentinel-2 imagery to a local machine for further processing.

*Atmospheric correction* Once the data is downloaded, the ACOLITE software is used to perform the atmospheric correction. This ensured that both training data and input data were corrected with the same algorithm. The ACOLITE is a suitable tool for atmospheric correction of coastal and ocean regions and its validity for use in marine-plastic detection has been previously investigated and verified^12,19^. Dark spectrum filtering is used to ensure consistency with the training data set and sun glint correction is also applied.

*Machine learning step for prediction and thresholding* Following the atmospheric correction, the ACOLITE outputs are combined into one large multi-banded GeoTiff file and input patches are created. These patches are then given to the MAP-Mapper network architectures to make predictions showing a probability of each pixel in an image being MD&SP. Therefore, thresholding is applied to produce binary classification outputs of “MD&SP” or “not-MD&SP”. The dataset is split via the 2:1:1 procedure into the train (694 patches), validation (328 patches), and test (359 patches) samples.

*Cloud and land masking* It has been previously shown that cloud masking reduces not only the presence of clouds in analysis but also the chance of miss-classification. Cloud and cloud shadow masking was implemented by integrating Python F-mask into the tool. F-mask takes raw Sentinel-2 data as an input and produces a mask with integer values of 0-5 representing (0) land, (1) water, (2) cloud shadow, (3) snow, (4) cloud, and (5) no observation, respectively. For the purpose of MAP-Mapper density mapping, water pixels are kept and all other pixels are then masked.

Despite having land pixels after the F-mask operation, the land class is not good enough. The main important reason behind this is that F-mask is a tool developed for land masking. Hence, their performance especially for border regions (coastal areas in this paper) was not as expected. This performance might be neglected in a less sensitive application but should be addressed in marine debris monitoring. Therefore, an additional land masking operation is also implemented by cropping a worldwide geospatial vector file to the ROI to apply more strict masking to promote better discrimination on land/sea borders. The output of the abovementioned process is then used to mask all land so that only ocean regions are kept for further analysis.

*MDM calculation and density map creation* In order to obtain MD&SP density maps, we first obtain probability (direct network output) and detection (thresholded to become a binary output) maps. Each pixel coordinate is then converted to coordinate reference system points, consisting of a longitude and latitude where their corresponding probability and detection values are used to calculate a novel metric of *MDM* to visualise the MD&SP density.

*MD&SP density map visualisations* For each pixel coordinate, the calculated MDM values are then plotted on the map using hexagonal binning. Regardless of their area, for each test location, the width of a hexagon is set to 5km and for all plotted images each hexagonal area was fixed at approximately 22 km$^2$. Please note that the choice of 5 km/22 km$^2$ is empirical considering the varying sizes of each test location. For some locations such as Honduras, bigger hex sizes can also be chosen, but this time smaller scenes such as Hong Kong suffers from this choice. In order to make all the figures representative enough (subjectively) we decided on this choice for the purposes of this paper. The horizontal distance of each area is calculated by using the *haversine function*. This is then divided by 5 km—the width of each hexagon, and we obtain the horizontal hexagon number. This number is not always an integer thus we round the calculated number of hexagons into integers.

In order to better quantify the density mapping findings 50% left trimmed average MDM values are used for each hexagon whilst colour-coding the findings. Further detailed analysis of MDM values from each scene shows that at least 80% of the MDM calculations are 0.2 or lower for each test data set. Trimming the first 50% of the sorted MDM values only removes these small values in order to make the average MDM analysis consistent for the purpose of discriminating polluted hexagons from fewer ones. We would also like to emphasise that trimming only affects polluted region hexagons, and makes us highlight polluted regions in a proper way. Interested readers are suggested to choose smaller trimming levels for their research, but here in this paper, we would like to stick to the value of a descriptive statistic - which is the *median*, and thus selected %50 trimming level for MDM visualisations.

We used *mapbox* and *plotly* packages in Python to create density maps. We selected *OpenStreetMaps* option in Mapbox which renders maps and map tiles with the Web Mercator projection using the EPSG:3857 projected coordinate system (sometimes called EPSG:900913).

### MAP-Mapper network architecture

Starting from a generic U-net architecture, we performed several optimisation/improvement stages in order to develop the MAP-Mapper architectures such as changes in input channels, output channels, batch size, patch size, thresholding, and the number of feature maps in each layer.

The first novel improvement has been achieved by reducing the utilisation of all Sentinel-2 bands. Biermann et. al. FDI^18^ and NDVI indexes only require 4 of the spectral bands available in Sentinel-2 images. This suggests that some of the bands encode more salient information about material type than others. Also, other bands may be introducing noise as they may not correlate with material type. Thus, extra bands may increase computational complexity, without improving detection. For these reasons, MAP-Mapper machine learning architectures are trained with only 4 input Sentinel-2 bands of 4, 6, 8, and 11. In our initial tests, this provided promising results and was just as effective as a model trained with all bands. This significantly reduces (i) the amount of data required for MD&SP detection, and (ii) MAP-Mapper architectures’ run-time.

In architectures like U-net, feature maps of the first layer extract simple features whilst the feature maps in later levels extract increasingly complex features. Consequently, even though increasing the number of feature maps increases the number of model parameters, this typically increases the model’s ability to learn. On the other hand, complex models have a tendency to over-fit, take longer to train, and take longer to make predictions on input data. Therefore, a balance is required. The MAP-Mapper development stages showed us the highest number of feature maps among 64, 128, 256, and 512 for each corresponding encoder layer achieved the best performance, so thus set as the MAP-Mapper architecture parameter.

We found out that (parallel with the expectations) smaller batch and patch sizes were correlated with significantly more gradual and longer model training times. It was found that smaller patches and batch sizes resulted in better model performance. Therefore, MAP-Mapper architectures consist of a batch size of 64 and a window size of 32.

Binary classification requires a threshold to discriminate whether the class is MD&SP or not. In general, the decision boundary line was set at 0.5. Therefore, any pixel that is predicted to be MD&SP with a probability greater than 0.5 was classified as MD&SP. Thresholding changes this value to influence the recall and precision of a model. A precision-recall curve was created to enable the identification of the optimal threshold, which resulted in the threshold of 0.815 producing an $F_1$ of 88% with a precision of 0.87 and a recall of 0.88 (Threshold of the MAP-Mapper-Opt architecture).

Map-Mapper-Opt precision value of 0.87 can be seen as low for a reliable MD&SP monitoring application. This is because of concerns about false positives being detrimental to the validity of the results. Therefore, the threshold was increased further to achieve higher precision values. MAP-Mapper-HP achieves a precision of 0.95 with a threshold of 0.99 whilst still maintaining a reasonable recall value of 0.63 ($F_1$ of 75%).

There is one clear consequence that users could choose one setup over another. MAP-Mapper*-HP* is designed to increase the detection of MD&SP pixels where other classes do not have importance for the purpose of the study. However, if detecting MD&SP is equally important in a multi-class detection problem, in this case, the MAP-Mapper*-Opt *version should be chosen over *-HP* since it is optimised to maximise the $F_1$ score via weighting false positives and false negatives equally.

## Data Availability

The Python code for the MAP-Mapper software v.1.0 has been published on the GitHub page https://github.com/CoDIS-Lab/MAP-Mapper. Further updates of the software will follow.
